# Torpor on Demand: Heterothermy in the Non-Lemur Primate *Galago moholi*

**DOI:** 10.1371/journal.pone.0010797

**Published:** 2010-05-24

**Authors:** Julia Nowack, Nomakwezi Mzilikazi, Kathrin H. Dausmann

**Affiliations:** 1 Department of Animal Ecology and Conservation, Biocentre Grindel, University of Hamburg, Hamburg, Germany; 2 Department of Zoology, Nelson Mandela Metropolitan University, Port Elizabeth, South Africa; University of Queensland, Australia

## Abstract

**Background:**

Hibernation and daily torpor are energy- and water-saving adaptations employed to survive unfavourable periods mostly in temperate and arctic environments, but also in tropical and arid climates. Heterothermy has been found in a number of mammalian orders, but within the primates so far it seems to be restricted to one family of Malagasy lemurs. As currently there is no evidence of heterothermy of a primate outside of Madagascar, the aim of our study was to investigate whether small primates from mainland Africa are indeed always homeothermic despite pronounced seasonal changes in weather and food availability.

**Methodology/Principal Findings:**

One of the nearest relatives of Malagasy lemurs, the African lesser bushbaby, *Galago moholi*, which inhabits a highly seasonal habitat with a hot wet-season and a cold dry-season with lower food abundance, was investigated to determine whether it is capable of heterothermy. We measured skin temperature of free-ranging individuals throughout the cool dry season using temperature-sensitive collars as well as metabolic rate in captured individuals. Torpor was employed by 15% of 20 animals. Only one of these animals displayed heterothermy in response to natural availability of food and water, whereas the other animals became torpid without access to food and water.

**Conclusions/Significance:**

Our results show that *G. moholi* are physiologically capable of employing torpor. However they do not use it as a routine behaviour, but only under adverse conditions. This reluctance is presumably a result of conflicting selective pressures for energy savings versus other ecological and evolutionary forces, such as reproduction or territory defence. Our results support the view that heterothermy in primates evolved before the division of African and Malagasy Strepsirhini, with the possible implication that more primate species than previously thought might still have the potential to call upon this possibility, if the situation necessitates it.

## Introduction

Hibernation and daily torpor are traditionally seen as energy-saving adaptations to survive unfavourable periods in temperate and arctic climates. However, heterothermy is also known to occur in mammals living in tropical and arid habitats and has been observed in Australasian, Neotropical and Afrotropical animals [1], [2], [3], [4], [5], [6], [7], [8], [9], [10], [11], [12]. Heterothermy has been found in a number of mammalian orders. Whereas in some groups a large number of species show heterothermy [13], within the primates it seems to be restricted to one family of small (30–500 g), nocturnal Malagasy lemurs, the Cheirogaleidae (incidence is confirmed in *Microcebus murinus*, *M. berthae*, *M. ravelobensis*, *M. rufus*, *Mirza coquereli* and *Cheirogaleus medius*, and suspected in most others) [4], [5], [7], [8], [11], [14]. Since the Cheirogaleidae are the most primitive of the lemur families, and heterothermy is so widespread within this family, it seems likely that the first primate inhabitants of Madagascar also exhibited this trait. Furthermore the capacity of lemurs to use heterothermy is believed to be a prerequisite for the successful colonization of Madagascar by lemurs from mainland Africa via rafting [15], [16]. Accordingly, torpor should be a plesiomorphic character and heterothermy should also be found in mainland relatives (Lorisiformes: Lorisidae & Galagidae), if it was not secondarily lost. However, there is no published evidence of heterothermy of a primate outside Madagascar.

At present, there are only three thermoregulatory studies on the closest mainland relatives of lemuriforms, which could not confirm the use of heterothermy: Müller et al. (1985) found that slender loris, *Loris tardigradus* (Southeast Asian Lorisidae) cool large parts of the body during cold exposure but still keep the body core at a high temperature under laboratory conditions [17]. A study on captive-bread African lesser bushbabies, *Galago moholi* (Galagidae) by Knox and Wright (1989) found a high degree of homeothermy when the animals were exposed to temperatures ranging from 6°C to 35°C in the laboratory for less than three hours [18]. Similarly Mzilikazi et al. (2006) did not find any incidence of heterothermy in *Galago moholi* in the wild [19]. This is especially surprising, as *Galago moholi* is one of the closest relatives of Malagasy lemurs and one of the most likely candidates within the primate group to show hypometabolic states. It is a small (∼200 g), nocturnal primate, that lives in dry woodlands of South Africa, as well as in the region from Angola to Tanzania in a highly seasonal habitat with a hot wet-season and a cold dry-season with lower food abundance [19]. The results from the study by Mzilikazi et al. (2006) raise the question of how *G. moholi* manages to cope with the high seasonality of its habitat, especially as females of *G. moholi* give birth to twins once or twice a year (January–February and September–November) and gestation mainly takes place during the most energy demanding winter period. However the Mzilikazi at al. (2006) study only measured body temperature (T_b_) and no metabolic rate (MR) or other ecological, ethological or physiological parameters were measured to determine possible seasonal adjustments. Recent studies also indicate that physiological traits are not as fixed as previously thought and physiological parameters can be highly variable and can differ not only between closely related species, but also between different populations of one species, within populations (e.g. between sexes), and even for one individual under different conditions [20], [21], [22].

The aim of our study was to investigate whether *G. moholi* does indeed never employ heterothermy as a strategy to survive unfavourable periods. Our results show that *G. moholi* is physiologically capable of employing torpor, but does use it only under especially adverse conditions.

## Results

### Ambient conditions

During the study period, climatic conditions were typical for a semi-arid seasonal habitat during the austral winter. Ambient temperature (T_a_) was low during the night, but fairly high during the day. The lowest T_a_ during the study period was −4.1°C in July (mean: 5.2±5°C) and the warmest day was recorded in March with a maximum temperature of 40.8°C (mean: 25.2±5.1°C). The amplitude of daily variations in T_a_ varied between a minimum value of 2.8°C and a maximum value of 29.9°C (mean: 20.0±4.7°C). The coldest month was July with the lowest minimum and maximum T_a_ of the study period. During the whole study period it rained on twelve out of 148 days. The average rainfall was 15.5 mm (total: 201.5 mm). Most rainy days occurred in March; however it also rained on two days during midwinter.

### Incidences of heterothermy

Torpor bouts were observed in autumn (April) and in the middle of winter (July). Torpor occurred in one free-ranging animal, as well as in two animals in the laboratory (during metabolic measurement). All three animals went into torpor in the early morning hours (6am–7am) and stayed torpid for four to six hours. Minimal skin temperature (T_skin_) varied between 21.8°C and 25.9°C (mean: 24±1.7°C).

**Figure 1:**
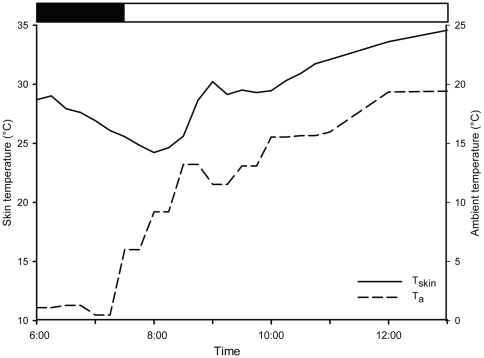
Skin temperature profile of a sub adult male *G. moholi* in winter including a torpor bout. Skin temperature (T_skin_) started dropping in the morning at 6am. The male was torpid for about four hours until 10am. *Black line* shows T_skin_, *dashed line* shows ambient temperature (T_a_). *Black bar* indicates the dark phase, *white bar* indicates the daytime.

Only one animal entered torpor under free ranging conditions multiple times. Torpor in this sub adult (sb) male (165 g) was recorded on six out of nine monitored days under unrestricted free-ranging conditions during the coldest month (between the 4^th^ and 19^th^ of July 2009, after which transmitter signal could no longer be detected). In all cases the animal went into torpor in the early morning hours and was normothermic again between 10am and 12am. The minimum T_skin_ recorded for this animal was 24.2°C (T_a_ 9.2°C) during a four hour torpor bout (6am until 10am) (fig. 1).

**Figure 2:**
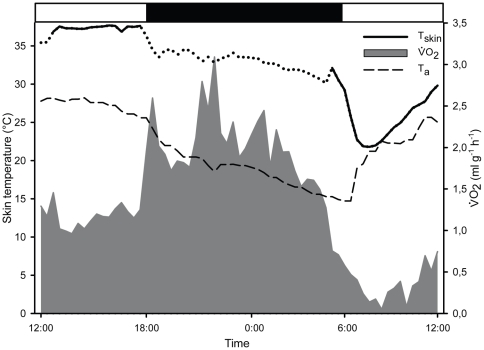
Metabolic rate and skin temperature of a sub adult female *G.moholi* including a torpor bout. Skin temperature (T_skin_) started dropping below 30°C in the morning at 5:30am; Metabolic rate (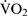) started decreasing earlier at 4am. The female was torpid for about six hours. *Grey area* illustrates 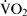, *black line* shows T_skin_, *dotted line* indicates the period of artificial variations in T_skin_ measurements due to movements of the animal, *dashed line* shows ambient temperature in the laboratory room (T_a_); *black bar* indicates the dark phase, *white bars* indicate the daytime.

Two out of twelve animals (female, sb; juvenile with uncertain sex) entered torpor during MR measurement in the laboratory, presumably as a reaction to food and water restriction. Both incidences occurred between the 12^th^ and 16^th^ of April 2009. The minimum T_skin_ recorded was 21.8°C (female, 180 g) closely reflecting T_a_ and 25.9°C (juvenile, 95 g). MR started decreasing about 60 min before T_skin._. For the female, the minimal oxygen consumption (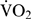) during torpor was only 1/10^th^ (0.09 ml g^−1^ h^−1^, T_skin_ 22.8±1.8°C, T_a_ 22.2±0.7°C) of minimal 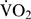 during normothermic resting conditions (0.99 ml g^−1^ h^−1^, T_skin_ 35.3±0.7°C, T_a_ 28±0.2°C) (fig. 2). The torpor bout was initiated around 5:30am and was terminated by the animal around 12pm. The energy savings of the juvenile during torpor were not as pronounced, with 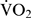 decreasing down to a value of 0.4 ml g^−1^ h^−1^ (T_skin_ 26.4°C±0.5°C, T_a_ 23.3±1.5°C), which was a 32% reduction of energy expenditure (1.23 ml g^−1^ h^−1^ under normothermic resting conditions, T_skin_ 34.4±0.3°C, T_a_ 27.3±0.4°C). This animal was disturbed before it spontaneously terminated its torpor bout after about 4.5 hours at 11pm with a T_skin_ of 27°C.

All other animals remained normothermic during metabolic rate measurements as well as under free-ranging conditions. Mean 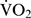 of normothermic animals (N = 10) under resting conditions in the laboratory was 0.96±0.25 ml g^−1^ h^−1^ (T_skin_ 33.9±1,5°C, T_a_ 23.6±3.3°C) and 2.79±0.52 ml g^−1^ h^−1^ (T_a_ 19.4±2.3°C) during the activity phase.

In contrast to the metabolic measurements under laboratory conditions no incidence of torpor was recorded during metabolic measurements inside the enclosure.

## Discussion

Our study presents the first confirmation of heterothermy in a primate outside Madagascar, showing that *G. moholi* does indeed have the ability to employ torpor and thereby decrease energy expenditure by up to 90%. In contrast to the Malagasy lemurs where most individuals enter torpor or hibernation at some point during winter only a very small proportion of the Galagos became torpid. In fact, only one animal was torpid under undisturbed, free-ranging conditions. The other animals became torpid in the metabolic chamber, presumably in response to food and water deprivation over a 24-hr period. Evidently, *G. moholi* has the potential to enter torpid states, but probably only does so under especially adverse conditions, so torpor bouts are relatively rare. These results, together with earlier studies on free living *G. moholi* that did not find any incidence of torpor [19], demonstrate the potential flexibility of physiological parameters within one population. Physiological plasticity with regard to thermoregulatory adaptations was found to depend on T_a_ and body weight which might also be the case for *G. moholi* [4], [20], [23]. All animals found to use heterothermy in this study were either sub adults or even born within the study year. Therefore, torpor seems to be restricted to or at least more common in younger *G. moholi*. Adult *G. moholi* might either be better adapted to cope with unfavorable conditions such as cold climate and food scarcity (e.g. because of their greater body weight), or they can simply not afford to become torpid, e.g. not to compromise reproduction or territory defense.

The advantages associated with the use of torpor, mainly the conservation of energy and water, are well understood [13] whereas the potential ecological and evolutionary forces constraining the use of torpor remain unclear. In *G. moholi* the wide-spread torpor avoidance may be associated with its breeding pattern [19]. For females it might be disadvantageous to enter torpor during their first pregnancy in winter because of a delay of foetal development as found in bats [24], [25], [26]: a shift of the first births would cause a shift of the second mating season to later in the year, which may be too late for the second litter to develop enough before the next winter to survive the cold and food restricted period. We therefore suggest a trade-off situation in the possibility to save energy and water by entering torpor versus the possibility of producing a second litter.

Males, on the other hand, might struggle to defend their territories during winter when undergoing torpor and therefore will have a lower mating success during the subsequent mating season in October. As territoriality is known to be correlated with high testosterone levels it is likely that torpor use during winter is precluded by a high testosterone concentration in the blood of the males. Behavioural observations have indeed shown that male *G. moholi* have overlapping home ranges that are defended throughout the night during winter (pers. obs. JN).

Up to now, heterothermy within primates was thought to be restricted to Malagasy lemurs, [4], [5], [7], [8], [20], [27] raising the question if this trait may have evolved in this group to cope with the especially challenging and unpredictable habitat conditions of Madagascar [28] or if it is a plesiomorphic character that enabled mainland Strepsirhini to colonize Madagascar [15]. Our study provides the first evidence of heterothermy in a primate outside Madagascar, thus suggesting that this trait may have evolved in this group before the division of African and Malagasy Strepsirhini, with the possible implication that more primate species than previously thought have the potential to use heterothermy to reduce energy and water expenditure. This finding supports the idea that adaptive heterothermy is a plesiomorphic characteristic in mammals and birds [6], [29], [30], [31].

## Materials and Methods

### Ethics statement

All procedures in this study complied with the “Principles for animal care”, publication no. 86–23, revised 1986 (National Institute of Health) and the “Code of ethics for animal experimentation” manual adopted by the Nelson Mandela Metropolitan University (animal ethics clearance no. A09-SCI-ZOO-001) and all experiments comply with the current laws of the country where they were performed. Animals were captured under permit no. CPM-002-00003, issued by the Department of Economic Development, Environment and Tourism.

No injured animals, lactating females or juvenile animals under the weight of 95g were used for metabolic measurements. Animals used in metabolic measurements were monitored at regular intervals to ensure animal welfare. Most animals were released after 24 hours, but no animal was kept longer than 84 hours (metabolic measurements in the enclosure) in captivity.

### Study site

The field work described in this study was conducted at the Nylsvley Nature Reserve (South Africa, Limpopo, 24°38.802′ 28°40.095′, altitude: 1100m), which is a semi-arid, mixed bushveld habitat. The hot wet season lasts from October to March, and the cool, dry season from April to September. The reserve receives a mean annual precipitation of 630 mm. Monthly mean winter temperatures range between −0 and 3.5°C [32]. The annual mean temperature is 19°C and monthly mean temperature ranges between −3.2 and 23°C [33].

We captured 23 animals from March to August 2009 using self-made walk-in live traps baited with bananas, honey and peanut butter. The traps were set late in the afternoon and checked early in the morning. Animals were classified in age according to teeth abrasion and reproductive status into one of three categories: adult (ad) (teeth blunt, testes or nipples visible), sb (teeth sharp, testes visible, but no nipples visible in females) or juvenile (weight <150 g, no testes or nipples visible). Animals were sexed, measured, weighed, individually marked with subcutaneously injected passive identification transponders (ID100 Trovan, EURO I.D. Usling GmbH, Weilerswist, Germany) and equipped with collars for temperature measurements (see below). Some of the animals were briefly anaesthetized with Ketaminhydrochloride (Ketanest® 1 mg/100 g, Parke-Davis, Berlin) for handling.All animals were released at exact capture locations.

### Skin temperature

T_skin_ of free-ranging animals was measured using collar temperature loggers (Weetags, 2.6g; Alpha Mach Inc., Mont St-Hilaire, Canada) and temperature-sensitive collar transmitters (TW-4 button cell tags, 4g; Biotrack, Wareham, UK). We equipped 20 of the 23 animals with Weetag loggers (resolution: 0.0625°C) which were programmed to measure and store T_skin_ automatically once an hour, so that these data loggers could log T_skin_ for about three months. We recaptured and obtained data from thirteen animals from March to July 2009 and time spans of data collection varied between ten days and 100 days. For improved precision, calibration curves provided by the manufacturer were used (calibration from 5°C to 30°C). On six animals, we used temperature-sensitive collar transmitters (three of them were fitted with Weetag loggers prior to the collar transmitters). These change their pulse rate according to temperature. The temperature-sensitive transmitters were calibrated in a water bath using a digital thermometer as a standard (testo 700, Lenzkirch, Germany). The temperature range for calibration was chosen due to the expected T_b_ of the animals (4°C to 40°C, steps of 4°C). Transmitter signals were detected using a TR-4 receiver (Telonics, Inc., Mesa, Arizona, USA) with a flexible two-element yagi antenna. Data from transmitter collared animals were collected as and whenever possible. The use of collar loggers and transmitters avoided implantations. As *G. moholi* is a small animal that shows the typical curled-up body posture during the resting phases, with a collar logger or transmitter positioned inside and firmly pressed against the ventral surface, T_skin_ during sleeping phases is a reasonable approximation of core T_b_ [32], [33], [34]. Our data were accurate when the animals were curled up in a sleeping position (as during a torpor bout). The mean normothermic T_skin_ of curled up animals during rest was 35.2±2.3°C (N = 18) and therefore well within the range of core T_b_ reported in literature (34.8°C to 38.6°C, [19]). However during activity the temperature measurement was not as accurate and T_skin_ data of active animals have not been considered for further analyses. To differentiate between these artificial fluctuations and true decreases of T_skin_ due to the initiation of torpor bouts, animals were defined to have entered torpor when T_skin_ showed a distinct and stable decrease (<30°C) for longer time periods (>2hours) (e.g. see fig. 2). Termination of a torpor bout was defined as the moment T_skin_ increased above 30°C. This cutoff point between normothermy and torpor was chosen, because Mzilikazi et al. [19] never measured core T_b_ below 33°C.

### Metabolic rate

Measurements of MR were conducted in a laboratory as well as within an outside enclosure. In both cases the animals were placed in the enclosure in the morning of the capture day and were left undisturbed for at least one hour before the beginning of the measurements.

Energy expenditure was determined by measuring rate of 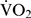 with open flow-through respirometry using a portable oxygen analyzer (FoxBoxC, Sable Systems International, USA). The metabolic chamber was connected to the FoxBox with airtight tubes and air which was dried with silica gel prior to analysis, was pumped through the system (pull mode). Flow rates were adjusted so as to maintain <1% oxygen depletion between incurrent and excurrent air (about 500 ml min^−1^ in the enclosure and between 900 ml min^−1^ (rest) and 1400 ml min^−1^ (activity) in the laboratory). CO_2_ was scrubbed prior to analysis of 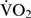 using soda lime and silica gel. 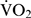 was calculated using the data acquisition program Expedata (Sable Systems International, USA) and the equation by Withers, 1977 [35]. The use of a multiplexer (TR-RM8, Sable Systems International, USA) allowed for automatic switching between an animal channel (15 min and 30 min in the laboratory and enclosure, respectively) and a reference channel (7 min and 15 min in laboratory and enclosure, respectively). For each 15 min or 30 min measurement, the mean value was calculated from 33% of the values (sampling frequency every 10 sec) which represented the most stable readings in the cycle. 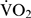 values for the resting MR were computed by using the mean of the three lowest consecutive values recorded during the entire rest phase. Similarly, active MR was calculated from the mean of the three highest values recorded during the active phase. The resting phase was defined as the time frame from 6am to 6pm due to behavioural observation (pers. obs. JN).

During all measurements of MR T_skin_ was measured simultaneously with Weetag collars in the laboratory that where programmed to measure T_skin_ every minute, and temperature-sensitive radio collars in the enclosure for real time monitoring. Ambient conditions were measured with the help of humidity and temperature loggers (thermochron iButtons, Maxim, USA) every 30min (resolution temperature: 0.0625°C, resolution humidity: 0.04%).

### Metabolic measurements in the laboratory

Measurements of MR in the laboratory were performed from end of March until end of April. To reflect the prevailing temperatures at the study site windows were kept open during measurements. MR was measured from a plastic metabolic chamber (30×15×18 cm, 8 l) for 24 hours in twelve wild *G. moholi* (6 males (4ad, 2sb), 5 females (2ad, 3sb), 1 juvenile animal, sex uncertain). The animals did not have any access to food or water throughout the measurements.

### Metabolic measurements in the enclosure

Measurements in the enclosure (180,5×61,5×193,5 cm) were performed from end of April until end of June under natural ambient conditions. MR was measured in nine wild *G. moholi* (4 males (2ad, 2sb), 5 females (2ad, 3sb)) during the resting phases (about 12 hours) of the animals on up to four consecutive days using a wooden nestbox (0.4 mm Plywood, 25×20×20 cm, 10l) as the metabolic chamber. During the night the animals were provided with water *ad libitum*, banana, gum of *Acacia* trees (if available) and mealworms.

### Ambient temperature

T_a_ was recorded within the animals' home ranges of both populations with thermochron humidity and temperature loggers every 30min. Data on rainfall was provided from the local weather station at Nyslvley Nature Reserve.
